# Evaluation of Root Canal Filling in Primary Teeth by Volumetric Analysis: *In Vitro* Study

**DOI:** 10.5005/jp-journals-10005-1545

**Published:** 2018-10-01

**Authors:** Prachi Sijeria, Rahul Bhartia, Nanjunda Swamy KV, Sadanand Kulkarni, Shilpy Singla

**Affiliations:** 1 Senior Lecturer, Department of Pedodontics, Rishiraj College of Dental Science, Bhopal, Madhaya Pradesh, India; 2 Senior Lecturer, Department of Conservative Dentistry and Endodontics, Rishiraj College of Dental Science, Bhopal, Madhaya Pradesh, India; 3 Professor, Department of Paediatric Dentistry, Sri Aurobindo College of Dentistry, Indore, Madhaya Pradesh, India; 4 Professor and HOD, Department of Paediatric Dentistry, Sri Aurobindo College of Dentistry, Indore, Madhaya Pradesh, India; 5 Professor and HOD, Department of Paediatric Dentistry, Rishiraj College of Dental Science, Bhopal, Madhaya Pradesh, India

**Keywords:** Bi-directional spiral, Cone beam computed tomography (CBCT), Lentulo spiral, NaviTip System

## Abstract

**Purpose:**

This study was conducted to evaluate the efficacy of various techniques to fill root canals of primary teeth by volumetric analysis using cone beam computed tomography (CBCT) to assess percentage of obturated volume (POV), percentage volume of voids (PVV), and depth-of-fill.

**Methodology:**

Root canals in 40 extracted deciduous maxillary incisors were instrumented with H-files to size 35 and volume of the canal measured using CBCT under “On Demand 3D AppTM Software” (Cybermed Inc. Medical 3D imaging software). The teeth were divided into four groups of 10 samples each and root filled by Lentulo spiral mounted on slow-speed hand-piece, NaviTip System, Bi-directional spiral and Combination method i.e. Lentulo spiral mounted on slow speed hand-piece followed by NaviTip syringe respectively, after that depth-of-fill for each group was checked. Then, the filled canal volume was measured using CBCT under “On Demand 3D AppTM Software”. Further, the (POV), (PVV) was calculated.

**Statistical analysis:**

The data were statistically analysed using one-way ANOVA, Turkey post Hoc test and Pearson’s Chi-square test.

**Clinical significance:**

Adequate adaptation of root canal filling material decreases the chance of microorganism regrowth, reinfection due to voids creation and minimizes the potential drawback of overfilling like foreign body reaction or deflection of the unerupted permanent tooth.

**Results:**

The four groups were comparable in canal volume. The overall percentage of obturated volume was 53%, 59.7%, 40.3% and 75.1%; the overall percentage volume of voids was 48%, 40.3%, 58.6%, 29.5%; optimally filled canals for each group was 80%, 60%, 30% and 90% respectively (p < 0.05).

**Conclusion:**

The greatest percentage of obturated volume and maximum number of optimally filled canals was obtained in method combining both Lentulo spiral in slow speed hand piece along with NaviTip system. Whereas, voids were the constant finding with all root fillings.

**How to cite this article:** Sijeria P, Bhartia R, Swamy KVN, Kulkarni S, Singla S. Evaluation of Root Canal Filling in Primary Teeth by Volumetric Analysis: *In Vitro* Study. Int J Clin Pediatr Dent, 2018;11(5):386-392.

## INTRODUCTION

The main objective of pulpectomy procedure in primary teeth is to fill the root throughout its length without gross over extension or underfilling^1^ and to avoid the creation of voids or gaps in the paste.^2-4^ The prognosis of pulp therapy in primary teeth depends on the quality of obturating material and obturation technique.^5-8^

Several approaches have been used to evaluate root canal filling quality *in vivo* and *in vitro.* The most common methods are conventional and digital radiography, but clinical radiographs are only 2-dimensional (2D) reproduction with difficulties in distinguishing feature superimposed onto each other.^9^ This study has used Cone Beam Computed Tomography (CBCT) for calculating the volume of root canal filling as it is an accurate and nondestructive method.

This *in vitro*-study was undertaken to make a 3-dimensional (3D) comparison of the filling quality of some most accepted filling techniques i.e. Lentulo spiral on slow speed handpiece (Dentsply Maille-fer),^10-12^ NaviTip system (Ultradent Products, Inc. South Jordon, Utah, USA)^10,13^ Bi-directional spiral (EZ-fill R EDSR USA)^14-16^ and a combination method i.e., Lentulo spiral along with NaviTip system to get the qualities of both the techniques in extracted primary maxillary incisors by volumetric analysis using CBCT with On-Demand 3D AppTM Software. The filling quality was evaluated based on the (POV), (PVV), and Depth-of-fill.

## METHODOLOGY

### Selection of Teeth

Forty extracted primary maxillary incisors were radiographed. Teeth with at least two third remaining root with no calcification or internal resorption and without any tooth anomalies were included. Soon the following extraction, teeth were kept in 0.5% sodium hypochlorite for one week for disinfection and then stored in distilled water. The sample was equally and randomly divided into four groups, i.e., 10 samples per group.

*Preparation of sample:* The teeth were decoronated at the cemento-enamel junction (CEJ) with a double-faced diamond disc to achieve a fixed reference point while doing CBCT analysis. The length of the roots was standardized 7 to 9 mm. Access opening was done and working length was taken by inserting a size 10 or 15 files into the canal until file existed from the apex; working length was set 1 mm short of the measured length. The BMP was carried out by H-file till size 35. 2 ml of 1% sodium hypochlorite and saline were used for irrigation.

**Figs 1A to D:**
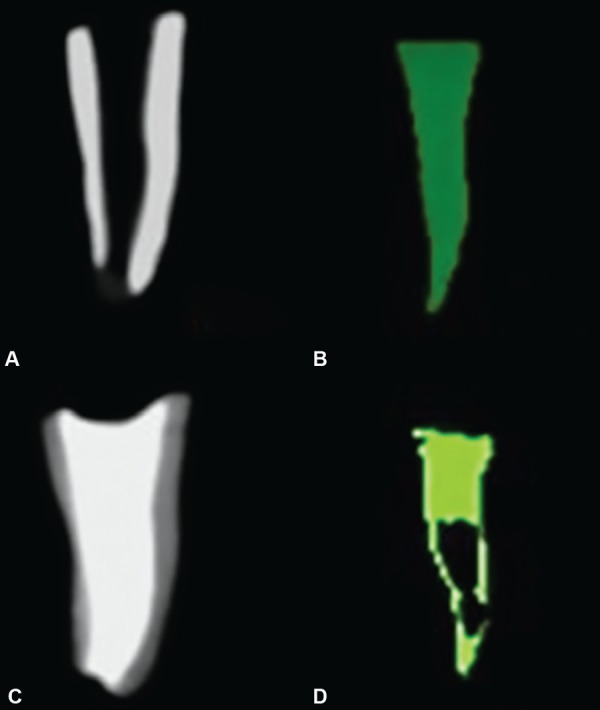
(A and B): Volume of canal before obturation traced green in color; (C and D): Volume of canal after obturation showing more void compared to group 2 and 4

**Figs 2A to D:**
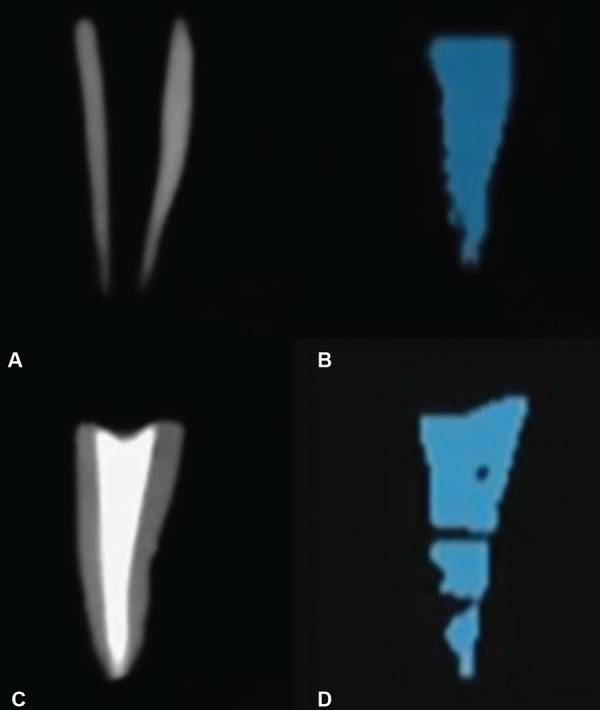
(A and B): Volume of canal before obturation traced blue in color; (C and D): Volume of canal after obturation showing more void compared to group 4

**Figs 3A to D:**
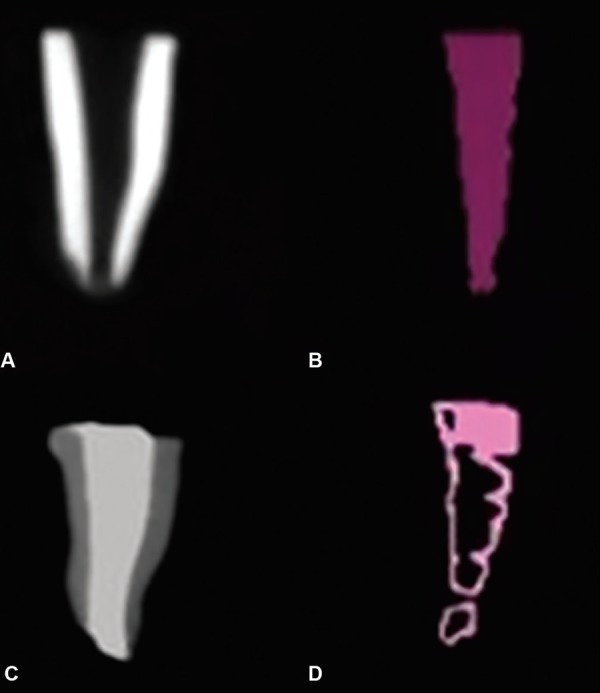
(A and B): Volume of canal before obturation traced violet in color; (C and D): Volume of canal after obturation showing maximum voids

**Figs 4A to D:**
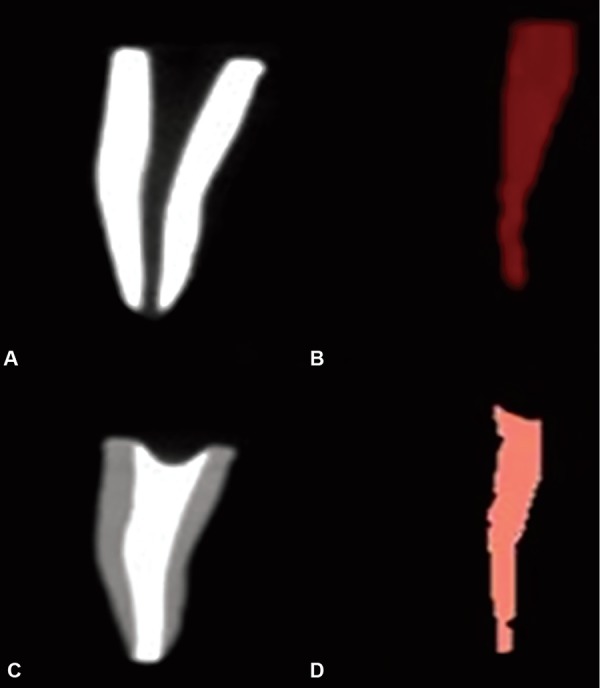
(A and B): Volume of canal before obturation traced red in color; (C and D): Volume of canal after obturation showing minimal voids

*Volumetric analysis before obturation:* 3D images of the roots were taken using the CBCT. The total volume of the root canal before obturation (X) was measured in cm^3^ using the “On Demand 3D AppTM software as shown in (Figs 1A and B, 2A and B, 3A and B and 4A and B.

*Root canal obturation:* After volumetric analysis, a small piece of paper was used to protect the apex followed by covering it with approximately 4 mm diameter ball of wax.12 the apical 1/3rd of the root was then embedded in a block 2 × 2 × 1 cm made up of sawdust mixed in plaster of Paris. After the plaster hardened, then to create an apical void the wax and the soft paper around the apex of each tooth was peeled off. To mimic the peri-radicular bone density, the created void was filled with a small ball of sponge, and then each tooth was suspended vertically.

The canals were then irrigated and dried using paper point to start with obturation. Zinc oxide and eugenol (ZOE) was the preferred material for obturation of primary teeth. As per the manufacturer’s recommendation and the technical limitation of obturation methods, a standardized mixture of pure ZOE without additive or fillers was prepared for each technique.^11^ After obturation, digital radiograph was taken for each sample to determine the depth-of-fill as underfilled, optimally filled and overfilled.^10,17^

**Group 1:** Lentulo spiral mounted on slow speed hand-piece

- *Group 1:* A Lentulo spiral instrument on a slow-speed hand-piece, one size smaller than the last size file was measured to the pre-determined canal length minus 1 mm. A rubber stop was placed around the thicker part of the spiral filler to reduce displacement during the filling procedures. The Lentulo spiral was dipped into the mixture and rotated into the canal. Additional amounts of paste were gradually introduced until the canal was filled.

**Group 2:** NaviTip system

- *Group 2:* A NaviTip system with 29-gauge and 29 mm length needle was selected. The needle was placed in the prepared root canals; a rubber stop was adjusted to the predetermined measurement. When back-fill of the paste from the canal orifice was observed, it was assumed that the canal was filled.

**Group 3:** Bidirectional spiral

- *Group 3:* A Bi-Directional spiral instrument was measured 3 mm short to the pre-determined canal length. The instrument was inserted into the canal in the forward direction (clockwise). Additional amounts of paste were gradually introduced until the canal was filled.

**Group 4:** Lentulo spiral followed by NaviTip system (combination method)

- *Group 4:* In combination technique (Lentulo spiral followed by NaviTip system), ZOE paste mixed to a creamy consistency, Lentulo spiral system was inserted into prepared root canal 2 to 3 times to achieve the greatest length of obturation followed by NaviTip system to get a dense fill with minimum voids. Once backfill of filling material occurred, the canal was assumed to be filled. A wet cotton pellet was used to pack the material inside the canal as a final finishing procedure.

For the volumetric analysis after obturation; new CBCT scans of the roots were done. The volume of filling material (ZOE) inside the canals were calculated in cm^3^ and recorded on a spreadsheet as final volume [Volume after obturation (Y)] as shown in Figs 1C and D, 2C and D, 3C and D and 4C and D.

Measurement of the volume of the canal before and after obturation allowed assessing the (POV) and (PVV). Data were analyzed statistically by one-way ANOVA; when one-way ANOVA showed significant results, Turkey post hoc test was applied for pair-wise comparison, Pearson’s Chi-square test was used to compare depth of fill among different techniques. Data analysis was done using Statistical Package for Social Sciences (SPSS) version 21 p < 0.05 was considered statistically significant.

## RESULTS

Table 1 shows the means and standard deviation of the volume of the canal (in cc) before obturation in each group. One way ANOVA showed no statistically significant difference (p = 0.367) among the four groups regarding the volume of canal before obturation.

One way ANOVA in Table 2 showed significant difference (p = 0.000) between the groups for percentage volume of obturation. When Turkey post hoc test was applied it showed that POV was significantly higher in group 4 (combination technique) than any other group in the study.

**Table 1:** **Table 1:** Comparison of volume of canal before obturation (in cc) in different groups

		*Volume before obturation (in cc)*							
*Groups*		*Mean ± SD*		*Min-max*		*One Way ANOVA*		*Tukey post hoc test (significant results)*	
Group 1: Lentulo spiral mounted on slow speed hand-piece		0.04 ± 0.01		0.03-0.06					
						1.087			
Group 2: NaviTip system		0.04 ± 0.01		0.02-0.07		p = 0.367			
Group 3: Bidirectional spiral		0.05 ± 0.01		0.03-0.07		(>0.05)		Not Applicable	
Group 4: Lentulo spiral followed by NaviTip system (combination method)		0.05 ± 0.01		0.03-0.06		Not Sig.			

**Table 2:** **Table 2:** Comparison of percentage of obturated volume in different groups:

		*Volume before obturation (in cc)*							
*Groups*		*Mean ± SD*		*Min-max*		*One Way ANOVA*		*Tukey post hoc test (significant results)*	
Group 1: Lentulo spiral mounted on slow speed hand-piece		53.00 ± 12.96		31-72					
						15.969			
Group 2: NaviTip system		59.70 ± 13.09		31-73		P = 0.000			
Group 3:Bidirectional spiral		40.40 ± 9.05		20-52		(< 0.05)		Gr4 > Gr1, Gr2, Gr3.	
Group 4: Lentulo spiral followed by NaviTip system (combination method)		75.10 ± 10.09		58-86		Sig. Diff.			

**Table 3:** **Table 3:** Comparison of percentage of volume of voids in different groups

		*Volume before obturation (in cc)*							
*Groups*		*Mean ± SD*		*Min-max*		*One Way ANOVA*		*Tukey post hoc test (significant results)*	
Group 1: Lentulo Spiral mounted on slow speed hand-piece		48.00 ± 11.65		32-69					
						15.969			
Group 2: NaviTip system		40.30 ± 13.09		27-69		P = 0.000			
Group 3:Bidirectional spiral		58.60 ± 9.65		48-80		(< 0.05)		Gr1, Gr2, Gr3 > Gr4	
Group 4: Lentulo Spiral followed by		25.90 ± 9.71		14-42		Sig. Diff.			
NaviTip system (combination method)									

**Table 4:** **Table 4:** Comparison of Depth-of-fill between different groups

*Groups*		*Optimally Filled Canals n (%)*		*Underfilled Canals n (%)*		*Overfilled Canals n (%)*		*Total n (%)*	
Group 1: Lentulo spiral mounted on slow speed hand-piece		08 (80.0)		00 (0.0)		02 (20.0)		10 (100.0)	
Group 2: NaviTip system		06 (60.0)		03 (30.0)		01 (10.0)		10 (100.0)	
Group 3:Bidirectional spiral		02 (20.0)		05 (50.0)		03 (30.0)		10 (100.0)	
Group 4: Lentulo spiral followed by NaviTip system (combination method)		09 (90.0)		00 (0.0)		01 (10.0)		10 (100.0)	

Pearson Chi Square Test value = 17.584, df = 6, p = 0.007 (< 0.05), Significant Difference

One way ANOVA in Table 3 showed a significant difference (p = 0.000) between the groups for percentage volume of voids. When Turkey post hoc test was applied it showed that PVV was significantly lower in group 4 than any other group in the study.

Pearson Chi-square test in Table 4 showed a significant difference (0.007) for a number of optimally filled canals, underfilled canals and overfilled canals between different groups. A maximum number of optimally filled canals is in combination technique followed by Lentulo spiral, NaviTip system, and Bi-Directional spiral respectively; maximum no. of underfilled canals is in a bi-directional spiral.

## DISCUSSION

Several objectives of pulpectomy procedures in primary teeth are considered; the one is there should be radiographic evidence of successful filling without gross overextension or underfilling.^1^ Another retrospective study showed two parameters as the highest predictor of final pulpectomy success: the pre-treatment pathologic root resorption and the quality of root canal filling relative to the apex.^18^

**Figs 5A andB:**
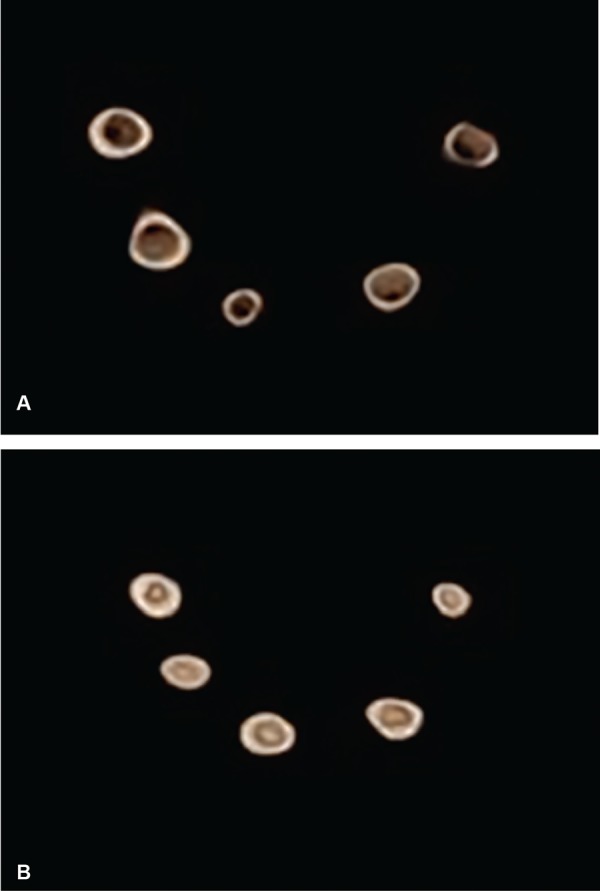
3D images of the roots before and after obturation

**Figs 6A and B:**
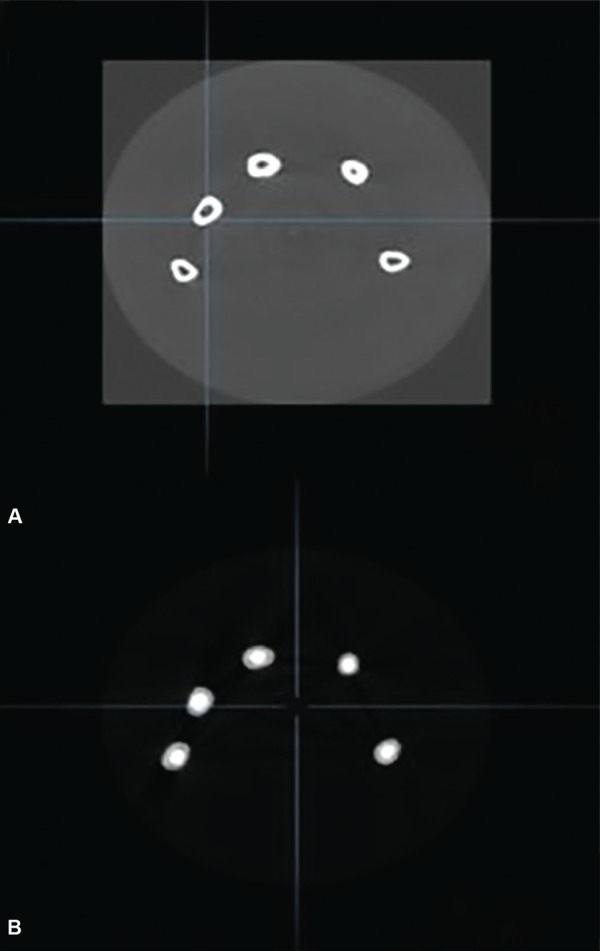
Axial images of the roots before and after obturation

Maxillary anterior were selected for this study as only straight canals were considered for standardization. The teeth were decoronated at the CEJ even though this procedure does not fully reproduce the clinical conditions.^19^ Decoronation allows specimen standardization by eliminating some variables, such as dental crown anatomy and the root canal access, thus providing a more reliable comparison of the proposed treatment techniques.^3^

As placement of the paste in the canal may influence the effectiveness of the filling procedure 20 therefore, various studies have compared different filling techniques *in vivo* and *in vitro.* But there is scant literature on CBCT evaluation of various obturation methods in primary teeth. Previously applied methodologies have many limitations, so to overcome those shortcomings, CBCT imaging was used in the present study (Figs 5A and B and 6A and B), as it is a non-invasive method which provides a 3D image of the morphological features.

The present study used On Demand 3D App™ software, to measure the total volume of interest in each canal in cm^3^. On Demand 3D App^TM^ includes all major modules (including 3D) and is ideal for an endodontist and other specialists. The segmentation tool in the software provides volumetric information, segment root canals using the density values and will provide the volumetric values.

The results of this investigation showed significant difference among the groups for a percentage of obturated volume (p = 0.000), percentage volume of voids (p = 0.000) and Depth-of-fill (p = 0.007).

Void was a constant finding with all the groups in the present study, this finding was similar to the previous reports,^6,13,21,22^ but the PVV differ with each technique. Root canal filling may provide pathways for leakage, mostly for voids present in the apical and coronal parts. Void also acts as a nidus for reinfection, leading to post-treatment diseases.^2^

The present study revealed that combination method (Figs 4C and D) led to the best filling quality regarding the highest POV, lowest PVV and a maximum number of optimally filled canals. Lentulo spiral is the most commonly used and one of the effective techniques. The design and flexibility of the Lentulo spiral spread the obturating material uniformly throughout, giving the greatest length of obturation.^10-12^ This is in agreement with the study by Aylard etal. and Dandashi etal., who concluded that the Lentulo spiral was a superior technique in obturating both straight and curved root canals of deciduous teeth.^11,12^ Torres etal., found that Lentulo spiral provides a homogenous fill throughout the canals compared to injection technique.^20^ However, to get a denser obturation NaviTip system was used following a Lentulo spiral in the present study.

The NaviTip system was specially designed to deliver paste into the root canal, and consists of a flexible tip.^2,10,18^ The highly flexible needle facilitates penetration into the curved and narrow canal close to the apex. It injects paste uniformly which gives a densely filled canal with minimum possible voids. The thin metal tip also increases operator feel during injection. This result is consistent with the studies by Guelmann et al. and Mahtab et al., who concluded that NaviTip syringe technique, minimize the chances of extrusion of paste out of the apex and produced the smallest size and lowest number of voids.^10,13^

Bi-directional spiral technique claims that a minimal amount of obturating material will past the apex.^14^ Present study showed the highest number of voids and underfilled canals. Grover et al. showed a similar result, who found maximum voids and the highest number of underfilled canals (75%).^22^

Studies showed, pulpectomies filled short or to the apex had a significantly greater success than overfilled canals^8,18,23,24^ due to the possibilities of extruding the ZOE beyond the root and initiating irritation.^8^ Whereas, Bawazir et al. reported that optimally filled and overfilled root canals showed significant radiographic success rates over underfilling.^17^ Nevertheless, overfilling should not be recommended over an optimally filled root canal. Potential drawbacks of overfilling are foreign body reaction or deflection of the unerupted permanent tooth.^24,25^

In the present study volumetric analysis was done for quality of filling, while in the previously reported studies voids were interpreted in two dimensions using conventional radiography.^13,22^ So, it is difficult to make a comparison among these studies. As clinical radiographs are only 2D reproductions, the radiographic monitoring of root canal treatment is challenging because of the difficulties in distinguishing features superimposed onto each other.^9^ Filling materials, dentine, cortical and trabecular bone, and soft tissues may mask voids in a root filling, even when using the theoretically optimum resolution.^9^

*Limitations and Recommendations for future research:* Further comparisons should be made with other techniques, with different types of teeth and canal configuration, or with other consistencies of ZOE and other materials for obturation. Clinical trials should be performed to ascertain the effectiveness of a combination technique in clinical practice settings. Another limitation is the operator sensitive technique operator’s skill need to be developed to obtain good results. Moreover, different thickness of the filling materials would have to be disregarded, due to the physical limitations of the different techniques.

## CONCLUSION

According to the proposed methodology and based on the finding of this study, the following conclusion may be drawn:

- Voids were the common finding with all the obturation technique. Therefore, none of the technique was able to provide a complete 3D fill.
- Combining two most accepted techniques, i.e. Lentulo spiral followed by Navitip system had the highest POV, highest no. of optimally filled canals and lowest PVV.
- The CBCT with On-Demand 3D software appears to be a valuable tool to locate voids and assess the efficacy of obturation.

## References

[1] (2004). American Academy of Pediatric Dentistry. Clinical guidelines on pulp therapy for primary and young permanent teeth. Pediatr Dent;.

[2] Peters CI, Koka RS, Highsmith S, Peters OA (2005). Calcium hydroxide dressings using different preparation and application modes: density and dissolution by simulated tissue pressure. Int Endod J.

[3] Agnol CD, Hartmann MS, Barletta FB (2008). Computed Tomography Assessment of the Efficiency of Different Techniques for Removal of Root Canal Filling Material. Braz Dent J.

[4] San S, Okte Z (2008). Success rate of Sealapex in root canal treatment for primary teeth: 3-year follow-up. Oral Surg Oral Med Oral Pathol Oral Radiol Endod.

[5] Richard M, Simcock, Hicks ML (2006). Delivery of Calcium Hydroxide: Comparison of Four Filling Techniques. J Endod.

[6] Fuks AB (2000). Pulp therapy for the primary and young permanent dentition. Dent Clin North Am.

[7] Cohen S, Hargreaves K (2002). Pediatric Endodontics: Endodontic treatment for the primary and young permanent dentition. Pathways of pulp. 8th edition.

[8] Yacobi R, Kenny DJ, Judd PL, Johnston DH (1991). Evolving primary pulp therapy techniques. J Am Dent Assoc.

[9] Sogur E, Baksi BG, Grondahl HG (2007). Imaging of root canal fillings: a comparison of subjective image quality between limited cone-beam CT, storage phosphor and film radiography. Int Endod J.

[10] Memarpour M, Shahidi S, Meshki R (2013). Comparison of Different Obturation Techniques for Primary Molars by Digital Radiography. Pediatr Dent.

[11] Aylard SR, Johnson R (1987). Assessment of filling techniques for primary teeth. Pediatr Dent.

[12] Dandashi MB, Nazif MM, Zullo T, Elliott MA, Schneider LG, Czonstkowsky M (1993). An in vitro comparison of three endodontic techniques for primary incisors. Pediatr Dent.

[13] Guelman M, McEachern M, Turner C (2004). Pulpectomies in primary incisors using three delivery systems: an in vitro study. J Clin Pediatr Dent.

[14] Musikant BL, Cohen BL, Deutsch AS (1998). Simplifying obturation. Dent Today.

[15] Parikh A, Banga KS, Thakore A (2000). Bi-directional spiral compared to traditional sealer placement techniques. Endodontology.

[16] Wu MK, Van der Sluis LW, Wesselink PR (2006). A 1-year follow-up study on leakage of single-cone fillings with Roeko RSA sealer. Oral Surg Oral Med Oral Pathol Oral Radiol Endod.

[17] Bawazir OA, Salama FS (2006). Clinical Evaluation of Root Canal Obturation Methods in Primary Teeth. Pediatr Dent.

[18] Coll JA, Sadrian R (1996). Predicting pulpectomy success and its relationship to exfoliation and succedaneous dentition. Pediatr Dent.

[19] Ferreira JJ, Rhodes JS, Pitt Ford TR (2001). The efficacy of guttapercha removal using Profiles. Int Endod J.

[20] Torres CP, Apicella MJ, Yancich PP, Parker MH (2004). Intracanal placement of calcium hydroxide: a comparison of techniques, revisited. J Endod.

[21] Subba Reddy VV, Shakunthala B (1997). Comparative assessment of three obturating techniques in primary molars: An in-vivo study. Endodontology.

[22] Grover R, Mehra M, Pandit IK, Srivastava N, Gugnani N, Gupta M (2013). Clinical efficacy of various root canal obturating methods in primary teeth: a comparative study. Eur J Paediatr Dent.

[23] Holan G, Fuks AB (1993). A comparison of pulpectomies using ZOE and KRI paste in primary molars: a retrospective study. Pediatr Dent.

[24] Kahn FH, Rosenberg PA, Schertzer L, Korthals G, Nguyen PNT (1997). An in-vitro evaluation of sealer placement methods. Int Endod J.

[25] Pinkham JR, Casamassimo PS, Mctigue DJ, Fields HW, Nowak AJ (2005). Pediatric Dentistry: Infancy through Adolescence..

